# Supplementary figures and images for: A stitch in time: The importance of water and sanitation services (WSS) infrastructure maintenance for cholera risk. A geospatial analysis in Harare, Zimbabwe
Source: PLoS Negl Trop Dis. 2023 Jun 16;17(6):e0011353. doi: 10.1371/journal.pntd.0011353 (PMC10275451; doi:10.1371/journal.pntd.0011353)

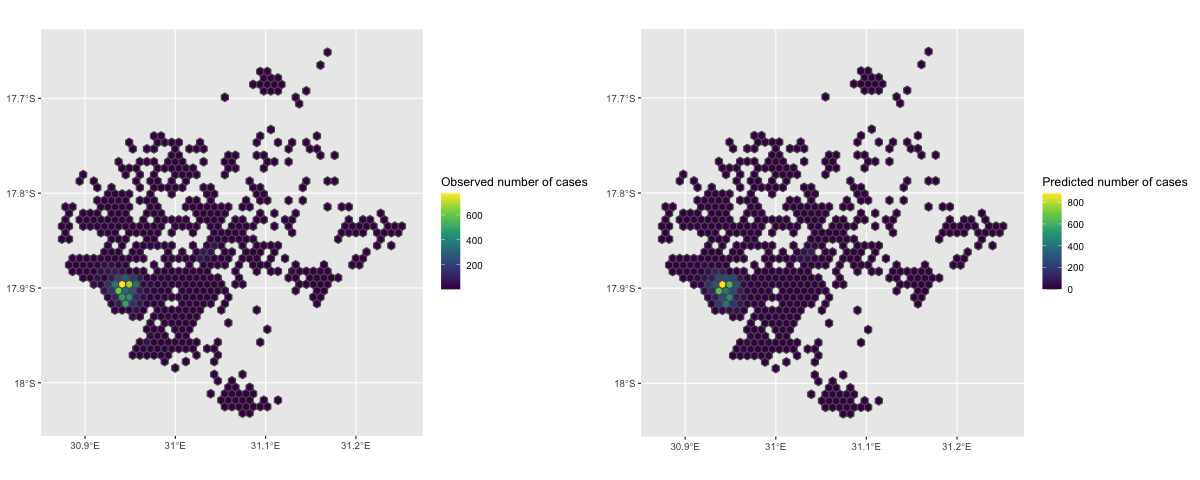

Supplement: S1 Fig — Map made with R package Leaflet v2.1.1 and map data from OpenStreetMap. (TIF) [file pntd.0011353.s001.tif]

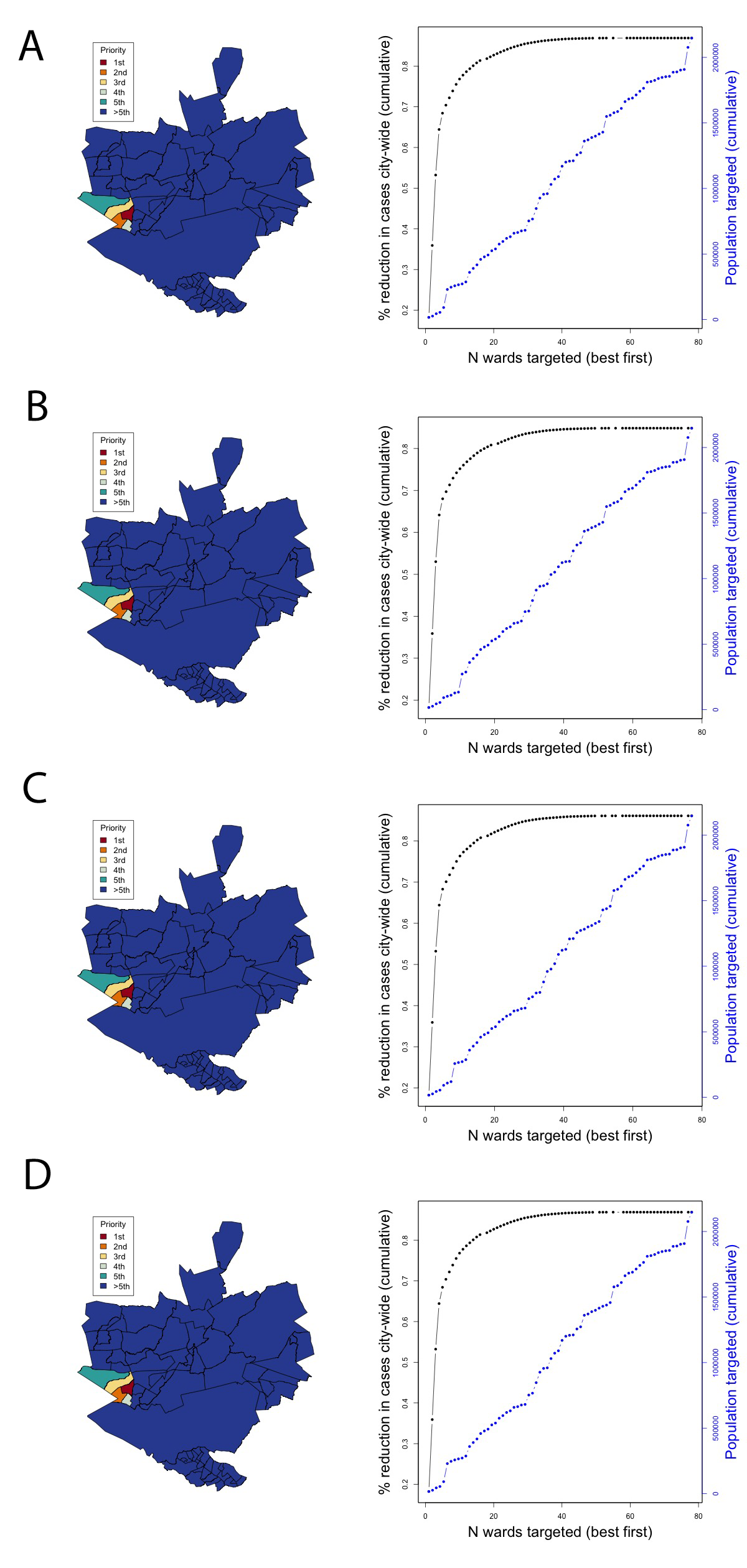

Supplement: S2 Fig — Left—The five wards in which targeting interventions would prevent the largest number of cases for the scenarios A–no sewer bursts, B–no sewer bursts within 200m of the water line, C—No sewer bursts in high (median) density burst areas and D—no sewer bursts in highest (75th percentile) density burst areas. Right–the cumulative reduction (%) in cases achieved, and population targeted, from targeting wards in order of priority. Base map attribution: CARTO, OpenMapTiles, OpenStreetMap contributors. (TIF) [file pntd.0011353.s002.tif]

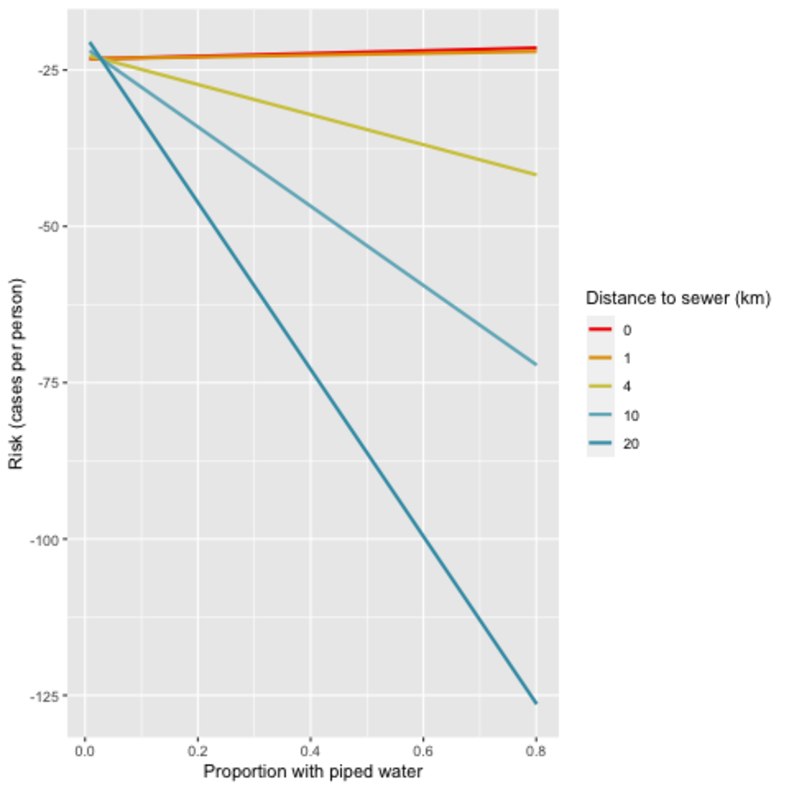

Supplement: S3 Fig — The y axis represents the risk (log of risk) when holding all other covariates at their mean value. Higher values indicate higher cholera risk. (TIF) [file pntd.0011353.s003.tif]
