# Supplementary material for: A stitch in time: The importance of water and sanitation services (WSS) infrastructure maintenance for cholera risk. A geospatial analysis in Harare, Zimbabwe
Source: PLoS Negl Trop Dis. 2023 Jun 16;17(6):e0011353. doi: 10.1371/journal.pntd.0011353 (PMC10275451; doi:10.1371/journal.pntd.0011353)
Supplement: Attachment — Submitted filename: PLOS_NTD_review6.21.22.docx [file pntd.0011353.s006.docx]

PLOS NTD Review 6.21.22

Global Comments

Please use past tense in the methods, as the study is completed.

Please appropriately number the tables in the text. Table 3 is listed as Table x.

Section on Scenario modeling – please improve punctuation throughout this section.

Minor Comments

Title: Please capitalize “Importance” in the title.

Abstract: Please clarify ‘human movement’. Is this human host movement, i.e. infected individuals alone or movement of both susceptible and infected individuals, i.e. the community?

Introduction: Please introduce the etiological agents of the diseases mentioned, cholera: *Vibrio cholerae*, and typhoid, *Salmonella Typhi*.

Last line, second paragraph: please clarify that infected and/or shedding host mobility is what is being assessed.

Third paragraph, first line. Please clarify this sentence. Do the authors mean, “In particular, outbreaks in developing country contexts are usually attributed to areas in which households have limited access to the WHO Joint Monitoring program. Specifically, where household water sources are classified as ‘improved’ in the delineation of water and sanitation infrastructure?”

Fourth paragraph, first line. Please change second use of risk to the word ‘likelihood’ for clarification.

Fifth paragraph, second line. Please spell out September and December.

Methods/Study Area: Please spell out 2008 – 2009.

Methods/Case Data: Please remove the subscript from the word epidemic, and place the footnote in the text, with a citation.

Methods/Case Data: Please spell out the word seven.

Results: Please add a citation to the improvement of modeling fitting for AIC.

Scenario Modeling: Please remove the superscript ‘2’ from the text and place the footnote in the paragraph with a citation.

Discussion: Please place an ‘a’ prior to the phrase ‘medium-sized market’.

Discussion: Please clarify what is meant by a ‘protective character.’

Discussion: Please remove the word ‘promises’ and replace with ‘the data suggests’.

Discussion: The statement, “there appeared to be limited benefits (in terms of reducing cholera risk) in investing in repairing sewer bursts throughout the city as it would have led to a .87 reduction in risk, versus a 0.86 reduction in risk when concentrating on the areas where the top 25% of sewer bursts are concentrated” is confusing and requires clarification.

Discussion: Please clarify this statement, “It is also more efficient to provide piped water to within 500 meters of the existing network (7.55), rather than to extend access to the whole city (8.2) when considering their relative impact on reducing the number of cholera cases.” It would be helpful to explain what the numbers are interpreted as, before suggesting infrastructure approach changes based on these numbers.

Discussion: Please change the statement, “we see” with ‘the data suggests’

Discussion: Please replace the word ‘perverse’ with ‘contradictory’.

Discussion: Please replace the word ‘who’ with ‘which’.
