# Supplementary material for: A stitch in time: The importance of water and sanitation services (WSS) infrastructure maintenance for cholera risk. A geospatial analysis in Harare, Zimbabwe
Source: PLoS Negl Trop Dis. 2023 Jun 16;17(6):e0011353. doi: 10.1371/journal.pntd.0011353 (PMC10275451; doi:10.1371/journal.pntd.0011353)
Supplement: Attachment — Submitted filename: Response to Reviewers Letter Form_221022_FINAL.docx [file pntd.0011353.s007.docx]

Dear Reviewers,

Thank you for taking the time to read and review our manuscript which we have renamed *“A Stitch in Time: The Importance of Water and Sanitation Services (WSS) Infrastructure Maintenance on Cholera Risk. A Geospatial Analysis in Harare, Zimbabwe”.* We greatly appreciate the thoughtful, constructive, and detailed feedback. We have worked to address all of the comments received. Please find below the editor and reviewer comments with our responses.

Thank you for your consideration and we will be happy to address any additional comments or feedback.

Reviewer's Responses to Questions

<b>Key Review Criteria Required for Acceptance?</b></br></br>
As you describe the new analyses required for acceptance, please consider the following:</br></br>

<b>Methods</b></br></br>
-Are the objectives of the study clearly articulated with a clear testable hypothesis stated?</br>
-Is the study design appropriate to address the stated objectives?</br>
-Is the population clearly described and appropriate for the hypothesis being tested?</br>
-Is the sample size sufficient to ensure adequate power to address the hypothesis being tested?</br>
-Were correct statistical analysis used to support conclusions?</br>
-Are there concerns about ethical or regulatory requirements being met?</br></br>

## Results

-Does the analysis presented match the analysis plan?</br>
-Are the results clearly and completely presented?</br>
-Are the figures (Tables, Images) of sufficient quality for clarity?</br></br>

## Responses:

Reviewer #3: The statistical approach used is not well described and no link to source code is provided. The authors cite a number of papers when describing their treatment of CDR data and the Poisson point process model but a formal description of what exactly they are doing is needed for me (and presumably most readers). Ideally the authors would also publish their data and code to allow for their results to be reproduced.

*Thank you for this comment. We have elaborated on the methods section of the paper and hope this now meets the requirements.*

*Source code can be found at* [*https://github.com/dime-worldbank/geospatial-cholera-modelling-zim*](https://github.com/dime-worldbank/geospatial-cholera-modelling-zim)

*With regards to data sharing, similar to the article we have found on Plos NTD by Qian et al (2022) (* [*https://doi.org/10.1371/journal.pntd.0010278*](https://doi.org/10.1371/journal.pntd.0010278)*) data that was collected from national and local government authorities (Ministry of Health, City of Harare, ZIMWA Catchment councils) is not suitable for public sharing. However, it can be made available on request to one of the authors by email to Faith Maidei Kashangura* [*fmashonga@gmail.com*](mailto:fmashonga@gmail.com) *who is Land Management and Development Manager in the Department of Works at City of Harare.*

*From the data sources listed in Annex 1, the following are available:*

| **Source** | **Data Type** | **Number of cases** | **Availability** |  |
| --- | --- | --- | --- | --- |
| City of Harare (all this data will only be available on request) | Cholera Cases | 9,890 cases | On request to [*fmashonga@gmail.com*](mailto:fmashonga@gmail.com). Dummy case data available in repo |  |
|  | Market and Public Facilities | 362 | On request to [*fmashonga@gmail.com*](mailto:fmashonga@gmail.com) |  |
|  | Water Bursts | 7,179 water reports | On request to [*fmashonga@gmail.com*](mailto:fmashonga@gmail.com) |  |
|  | Sewer Bursts | 24,713 sewer reports | On request to [*fmashonga@gmail.com*](mailto:fmashonga@gmail.com) |  |
|  | Boundaries of admin zones | 165 suburbs mapped | On request to [*fmashonga@gmail.com*](mailto:fmashonga@gmail.com) |  |
|  | Water Quality Sampling | 23 sample point names  matched with weekly water quality reports | On request to [*fmashonga@gmail.com*](mailto:fmashonga@gmail.com) |  |
|  | Water and sewer network | Shape files | On request to [*fmashonga@gmail.com*](mailto:fmashonga@gmail.com) |  |
|  | Public boreholes | 481 geo-coded | On request to [*fmashonga@gmail.com*](mailto:fmashonga@gmail.com) |  |
| Household Data (DHS, 2015) (can be shared) | Drinking water source/sanitation facility | 60 WASH clusters, 44 inside Harare | In repo <https://github.com/dime-worldbank/geospatial-cholera-modelling-zim> |  |
| Groundwater Specialist (available on request from World Bank) | Groundwater Vulnerability | 2 raster maps with groundwater risk level | On request to [gjoseph@worldbank.org](mailto:gjoseph@worldbank.org) or [sayling@worldbank.org](mailto:sayling@worldbank.org) |  |
| Manyame, Upper Mazowe, Nyagui Sub catchment Councils (available on request from these relevant catchment authorities) | Private Boreholes | 1848 private boreholes geocoded: 1,721 in Harare and 127 outside the city | On request via <https://zinwa.co.zw/> |  |
| FB HDX  (can be shared) | Population density | Raster | In repo <https://github.com/dime-worldbank/geospatial-cholera-modelling-zim> |  |
| World Bank from ZIMSTAT  (available on request from World Bank) | Poverty data | N/A | On request to [gjoseph@worldbank.org](mailto:gjoseph@worldbank.org) or [sayling@worldbank.org](mailto:sayling@worldbank.org) |  |

It isn’t clear how all the covariates in the models were decided upon. Was there a DAG or some theoretical model illustrating the hypothesis of associations to make sure simply throwing them all in and selecting based on AIC didn’t lead to unintentional confounding (e.g., collider bias)? The approach used seems reasonable if the aim is prediction but less ideal for inference about individual covariates (and scenarios).

*The covariates in the model were decided based on the known association between poor water and sanitation and transmission of cholera as is explained in the pathways to transmission in the introduction. “*Pathways for transmission can include contaminated water sources, both for drinking and domestic use [[5]](https://www.zotero.org/google-docs/?g5Mf0x)[[6], [7]](https://www.zotero.org/google-docs/?0F8EVu)[[8]](https://www.zotero.org/google-docs/?GfU6Nv), poor sanitation [[9]](https://www.zotero.org/google-docs/?9LHrwb)[[10]](https://www.zotero.org/google-docs/?uV3kEj)[[11]](https://www.zotero.org/google-docs/?vofexc); contaminated food, poor personal hygiene[[11]](https://www.zotero.org/google-docs/?ho7wgK) and poor drainage[[12], [13]](https://www.zotero.org/google-docs/?MhMDaI). Furthermore, spatio-temporal analyses have demonstrated how cases can cluster among close contacts and therefore be more prevalent in dense living environments where an uncontaminated water supply is not readily available for drinking, washing or cooking [[14]](https://www.zotero.org/google-docs/?SJ4zQu).” The scenario analysis has now been removed from the paper and we are presenting just the results of the point process modelling that shows spatial associations rather than inferring any causality.

To what spatial scale were pipe bursts etc digitised? Modeling is done at a pretty small scale and it would be good to make clear that there is reasonable certainty that digitisation of the water and sanitation infrastructure data are done at an appropriate scale. The authors should be even more clear in methods that this is just reports of pipe bursts but not necessarily confirmed pipe bursts.

*Pipe bursts were digitized using the cadastral map of Harare as has now been described in the paper. They were geocoded using the addresses provided by the customers who were reporting the bursts. Though they are reported bursts, the data also contains a field that denotes date and time of complaint redressal which implies that the sites of the complaints were visited and verified.*

Are sewer bursts time varying covariates in the model? If so, I think it would be helpful to understand the associations across different lags.

*Sewer burst data was made available to us from Dec 2017 – Dec 2019. Given that the outbreak was in January 2019, we cleaned the data and divided it into two time periods, before and after the outbreak. In the model, the accumulated density of bursts prior to the outbreak i.e. Dec 2017-Dec 2018 was taken as a covariate for the model.*

It would be helpful to describe which health facilities were included in the line list. Is there any risk that the locations of the primary cholera treatment centers influenced the observed spatial distribution of cases? Showing the health centers on a map along with cumulative incidence would be useful.

*As has now been added to the paper, given that the GPS locations of cases were directly mapped to the households using households reported addresses and the cadastral map, the health facility locations are unlikely to have biased results towards their own locations*.

It appears these data are from the MoHCC linelist. It would be helpful for the authors to better describe the at which admin level the cases were georeferenced to. Were there any missing data? How were these handled?

*Thank you for this question. The detail on the cholera case data has now been added to the paper. The line list was available at the individual level, with the locations of cases being plotted from cross referencing individuals’ home addresses with the cadastral map of the City of Harare. As stated in the paper “There were a total of 9,977 cases on the line list. Some of the cases had no addresses or had duplicate addresses and therefore could not be geocoded. Therefore, ultimately, 9,890 cases were included in the analysis after cleaning.” For privacy and ethical reasons, the case data itself is not being made available publicly but we are providing a dummy dataset for the methodology to be replicable on the github repository.*

For the WHO case definition provided, are areas where cholera is not known to be occurring neighbourhoods? Or is Harare treated as a unit and the suspected case definition is really just ≥ 2 y/o with AWD (except for the first case)?

*Thank you for the question. The full explanation of the procedure can be found at (*[*https://www.gtfcc.org/wp-content/uploads/2020/04/gtfcc-cholera-outbreak-response-field-manual.pdf*](https://www.gtfcc.org/wp-content/uploads/2020/04/gtfcc-cholera-outbreak-response-field-manual.pdf)*)*

***A cholera outbreak*** *is defined by the occurrence of at least one confirmed case of cholera and evidence of local transmission.*

*Laboratory confirmation by culture or PCR of the first suspected cases is essential to confirm a cholera outbreak.*

*• Collect stool specimens from the first suspected cases (5–10 cases) and send them to the laboratory for confirmation.*

*• Declare the outbreak if V. cholerae O1 or O139 is confirmed by culture or PCR with evidence of locally-acquired infection (exclude imported cases). The government can declare the outbreak with advice of the international agencies, local authorities and non-governmental organizations (NGOs).*

*• Once the outbreak is declared, there is no need to confirm all suspected cases. The clinical case definition is sufficient to monitor epidemiologic trends.*

*• Determine antimicrobial susceptibility patterns for the first isolates confirmed by the laboratory at the beginning of the outbreak to provide sufficient information to guide antimicrobial treatment.*

Water and Sanitation Infrastructure Data: These data are from what period of time? Same as the outbreak or something longer term?

*Thank you for the question. This has now been addressed in the data sources section of the paper*

*“The water and sanitation network shape files for the city were obtained with the support of the City of Harare’s (CoH) Water Department and Department of Works.The shapefiles were generated as part of an initiative that was started in 2012 to digitize all hard copies of CoH Water Department’s water and sanitation network structures. The process was completed in 2019 during which time no major infrastructure upgrades took place. Small edits were manually added in the digitized files.”*

It would be helpful to understand the marketshare of the two providers of CDR data to help the reader understand how representative these might be.

*Thank you for the question. This has now been addressed in the data sources section of the paper “We use anonymous and aggregated Call Detail Record (CDR) data provided by two Mobile Network Operators in Zimbabwe covering the period of the outbreak (September 2018-February 2019). These operators combined made up 76% of the market share in 2019 based on data from TeleGeography.”*

The following data availability statement is not consistent with PLOS’s data policy “Data is stored on the World Bank OneDrive. It can be accessed and shared in consultation with Harare City Council's Water utility. Cholera case data would have to be consulted with the Zimbabwean Ministry of Health and the WHO.” Data are supposed to be made open except in special circumstances (e.g., when they are identifiable).

*As given above:*

*With regards to data sharing, similar to the article we have found on Plos NTD by Qian et al (2022) (* [*https://doi.org/10.1371/journal.pntd.0010278*](https://doi.org/10.1371/journal.pntd.0010278)*) data that was collected from national and local government authorities (Ministry of Health, City of Harare, ZIMWA Catchment councils) is not suitable for public sharing. However, it can be made available on request to one of the authors by email to Faith Maidei Kashangura* [*fmashonga@gmail.com*](mailto:fmashonga@gmail.com) *who is Land Management and Development Manager in the Department of Works at City of Harare.*

*The reason is that some of this data, in particular the GPS coordinates of individuals households cannot be shared publicly as it would constitute a breach of privacy. For government data this would need to be shared more widely only following consultation with the relevant authorities.*

The authors find that almost all coefficients in their regression model do not have confidence intervals spanning the null. This is surprising to me given how rarely these variables have been associated with cholera incidence in the past and how small effect sizes tend to be. While I could be wrong (and can’t fully assess given the lack of detailed methods), it seems like this is an artefact of the methods used which dont appropriately account for correlated data.

*There are a number of reasons why we believe these statistical associations to be credible. Firstly, steps were taken to exclude highly correlated covariates before model fitting (as now described in the manuscript). Further, only covariates with a plausible association with cholera risk were included in the model. In addition, by leveraging 4000 quadrature points per timeslice (week), this increases the statistical power to detect small effect sizes*.

# Reviewer #3

## For Specific Comments:

In addition, it would be helpful for the authors to report interpretable coefficients (presumably exponentiated and perhaps with additional transformations). Hard to know how important these seemingly large effect sizes are.
- It is not clear to me how to interpret the results suggesting increases (e.g., 1e16!) in incidence from scenarios with sewer access provided to the entire city and those with piped water….

*As coefficients are in part determined by the units of the covariates themselves, we have decided to retain the coefficients as is to avoid uninterpretable rate ratios when exponentiated. We have added the following text to the Table 2 legend to further explain our decision.*

*‘Coefficients, showing the difference in the log of the expected counts for a unit increase in the covariate, are shown to avoid uninterpretable values when exponentiated to rate ratios.’*

Surprising findings from this work include the fact that proportion of those with unimproved water source is a protective factor and that increases in geological vulnerability is also a protective factor. The former can be explained by the proximity of piped water sources to sewers with a high number and density of bursts.” This may be the case but it also suggests that adjusting for bursts as done in these models is insufficient.

*We acknowledge that the analysis limits the certainty with which we can draw conclusions about the observed relationships. We have added the following to the discussion to provide a more speculative assessment:*

*It is unclear as to why those with a higher proportion of unimproved water are associated with lower cholera risk and may be explained by contamination of the piped water network which temporarily makes unimproved water sources relatively safer. In addition, there is undoubtedly error in the predicted proportion with unimproved water as these data are based on a small number of obfuscated DHS data points which could lead to a spurious relationship. Similarly, the relationship with geological vulnerability is difficult to interpret as the mechanisms of how geological vulnerability impacts the flow of pathogens and exposure to the population require further exploration.*

## Summary and General Comments

Use  this section to provide overall comments, discuss strengths/weaknesses of the study, novelty, significance, general execution and scholarship. You may also include additional comments for the author, including concerns about dual publication, research ethics, or publication ethics. If requesting major revision, please articulate the new experiments that are needed.

**Reviewer #2:** This paper uses geo-spatial analysis to explore the role of water and sanitation infrastructure on cholera risk taking case study of Harare, Zimbabwe. The findings of study are interesting. However, some findings such as proportion of those with unimproved water source is a protective factor and that increases in geological vulnerability is also a protective factor. The interpretation and justification of these findings is not convincing and need careful analysis and interpretation.

*Thank you for this comment, we have attempted to address this with the paragraph in the discussion section as described above:*

*“It is unclear as to why those with a higher proportion of unimproved water are associated with lower cholera risk and may be explained by contamination of the piped water network which temporarily makes unimproved water sources relatively safer. In addition, there is undoubtedly error in the predicted proportion with unimproved water as these data are based on a small number of obfuscated DHS data points which could lead to a spurious relationship. Similarly, the relationship with geological vulnerability is difficult to interpret as the mechanisms of how geological vulnerability impacts the flow of pathogens and exposure to the population require further exploration.”*

**Reviewer #3:** The manuscript “The importance of Maintenance: Geospatial Analysis of Cholera Risk and Water and Sanitation Infrastructure in Harare, Zimbabwe” presents results of a statistical spatio-temporal analysis exploring the associations between various potential risk factors related to the built environment, natural environment and socio-demographic factors and cholera incidence in Harare at a fine spatial scale. They then use the fitted regression model to explore the predicted incidence when setting covariates to different levels. The authors find associations between cholera incidence and several potential risk factors (far more than most studies find) and suggest that eliminating pipe bursts would have led to large reductions in cholera incidence. I think this is an interesting story but the authors do not explain their methods sufficiently to understand what exactly they are doing and from what I do understand, seem to be drawing very causal conclusions when at
best they are estimating (potentially biased) associations. This reads much more like a report than a manuscript and If this were to be published I would expect much more details, more sensitivity analyses and more restrained interpretation of the results.

Thank you for your thorough review of the paper. We have provided more detail on the methods section now, which we hope will provide clarity, as well as the source code and data being *uploaded to the github repository.*

*With regards to the overall tone and conclusions of the paper, we have attempted to articulate more clearly that these are associations and correlations rather than attempting to draw causal conclusions from the results. We have also removed the scenario analysis given the comments but hope that the reviewers can see the contributions of the research as worthy of publication. It draws upon uniquely detailed datasets to infer associations that can provide useful insights for policymakers on the spatial and infrastructure specific breakdown of cholera risk in the city.*

**Editorial Comments**

- Please use past tense in the methods, as the study is completed.

Addressed

- Please appropriately number the tables in the text. Table 3 is listed as Table x. Addressed
- Section on Scenario modeling – please improve punctuation throughout this section.

This section has been largely removed, but it is addressed

- Title: Please capitalize “Importance” in the title.
  - Addressed
- Abstract: Please clarify ‘human movement’. Is this human host movement, i.e. infected individuals alone or movement of both susceptible and infected individuals, i.e. the community?

Addressed – it refers to the population movement overall rather than infected individuals only

- Introduction: Please introduce the etiological agents of the diseases mentioned, cholera: Vibrio cholerae, and typhoid, Salmonella Typhi.
  - Added
- Last line, second paragraph: please clarify that infected and/or shedding host mobility is what is being assessed.
  - Addressed
- Third paragraph, first line. Please clarify this sentence. Do the authors mean, “In particular, outbreaks in developing country contexts are usually attributed to areas in which households have limited access to the WHO Joint Monitoring program.
  - Edited accordingly – instead it now says

“In particular, outbreaks in developing country contexts are usually attributed to where households have low access to ‘improved’ water and sanitation infrastructure such as networked water and sewer systems (as defined by the WHO Joint Monitoring program)”

- Specifically, where household water sources are classified as ‘improved’ in the delineation of water and sanitation infrastructure?”

Addressed above

- Fourth paragraph, first line. Please change second use of risk to the word ‘likelihood’ for clarification.
  - Addressed
- Fifth paragraph, second line. Please spell out September and December.
  - Addressed
- Methods/Study Area: Please spell out 2008 – 2009.
  - Addressed
- Methods/Case Data: Please remove the subscript from the word epidemic, and place the footnote in the text, with a citation.
- Methods/Case Data: Please spell out the word seven.
  - Addressed
- Results: Please add a citation to the improvement of modeling fitting for AIC.
  - Addressed on page 10, reference number 43, Hastie et al.
- Scenario Modeling: Please remove the superscript ‘2’ from the text and place the footnote in the paragraph with a citation.
  - This section has been removed, thus the comment is addressed
- Discussion: Please place an ‘a’ prior to the phrase ‘medium-sized market’.
  - Addressed
- Discussion: Please clarify what is meant by a ‘protective character.’
  - Reference to this language has been removed as it is misleading. It refers to a reduced association of that particular variable with cholera risk compared to others in the model and/or what is expected from the literature.
- Discussion: Please remove the word ‘promises’ and replace with ‘the data suggests’.
  - Addressed
- Discussion: The statement, “there appeared to be limited)
  - Unclear what the issue was but this has been changed to “there appeared to be relatively little”
