# Supplementary material for: A stitch in time: The importance of water and sanitation services (WSS) infrastructure maintenance for cholera risk. A geospatial analysis in Harare, Zimbabwe
Source: PLoS Negl Trop Dis. 2023 Jun 16;17(6):e0011353. doi: 10.1371/journal.pntd.0011353 (PMC10275451; doi:10.1371/journal.pntd.0011353)
Supplement: Attachment — Submitted filename: PLOS_NTD_12.8.22_Review.docx [file pntd.0011353.s008.docx]

**Global comments:**

Please use line numbers in the future.

Please double check spacing throughout the manuscript. Some areas are missing spaces, some are double-spaced where they should not be.

Please break down run on sentences.

Please spell out numbers from zero to nine.

Do not use symbols, i.e, < or >. Please spell out the words less than or greater than.

**Minor Comments**

**Abstract:** One run-on sentence should be broken down, instead reading as: One possible explanation for this observation is that sewer bursts led to the contamination of the piped water network. This turned access to piped water, usually assumed to be associated with reduced cholera risk, into a risk factor itself. Such events highlight the importance of maintenance in the provision of SDG improved water and sanitation infrastructure.

**Introduction:** Vibrio cholerae is the etiological agent of cholera, not the etiological agents. Also, serotypes O1 and O139 are responsible for causing cholera, as there are well over 200 serotypes characterized to date. Please specify this in the introduction, and cite.

Salmonella Typhi should be written as, “*Salmonella enterica* serovar Typhimurium”

The sentence, “In particular, outbreaks in developing country contexts are usually attributed to where households have low access to ‘improved’ water and sanitation infrastructure such as networked water and sewer systems (as defined by the WHO Joint Monitoring program) [3], [21]” is somewhat unclear. What is meant by contexts? Please clarify.

For the phrase developing country, it is usually more appropriate to utilize the term, “emergent nation”.

The phrase well documented should have a hyphen between the words.

**Study Area:** It is uncertain what the acronym DHS stands for. Please spell out prior to using the acronym in the paper.

**Case Data:** “Age and gender of the patients is” should be changed to “Age and gender of the patients are”

How many cases were duplicates that were removed? Please specify.

**Population Characteristics Data:**  What does the HDX stand for after Facebook? Please specify the acronym as requested previously.

“Household access to water, sanitation and hand washing infrastructure was taken from the DHS 2015.” Was should be changed to were.

The single sentence: “It placed households reporting their facility to be a flush toilet connected to a piped sewer system and not shared at the top of the ladder and therefore lowest risk, then flush toilets connected to a septic tank, pit latrine, VIP latrine or pit latrine with slab that were unshared; then each of the former facility types that were shared; then unimproved sanitation facilities; and finally households reporting open defecation as those with the highest sanitation risk.” Should be broken down into multiple sentences.

**Spatial modeling:**

The word “the” should be placed before Poisson.

The following sentence should read as, “For onsite facilities, contamination can occur through unlined or poorly constructed pit latrines which may seap content into the groundwater supply. Thus, groundwater vulnerability is also included in the model because it feeds into the many borewells in the city[41].”

The following paragraph should read as, “We include the market and public facility locations, as well as the population density data, due to the pathways to contamination that have been identified in contaminated food and poor personal hygiene [11]. In addition, cholera transmission has been associated with case clusters among close contacts respectively [14]. We finally include an indicator that accounts for mobility by calculating the expected imported cases that come in from other areas. Emigration could lead to the generation of additional cholera cases in an area, given research showing that population mobility can lead to spread of cholera to new areas [16], [18]–[20].

On page 8, please clarify the acronym SIM.

The following sentence should read as, “For every individual/SIM we calculated the probability that an individual traveling to a destination became infected in the last ten days. This calculation was based on the 1) locations the individual spent time in during those ten days, 2) duration of time spent in each location and 3) local level of risk at each location (estimated by calculating observed weekly incidence per tower catchment).

**Results**

All instances of the phrase, ‘the modeling’ should be changed to ‘the models.’

The sentence, “In addition, there is undoubtedly error in the predicted proportion with unimproved water as these data are based on a small number of obfuscated DHS data points which could lead to a spurious relationship.” Is unclear. This seems like an important point, please expand, but be careful not to jump to conclusions without data to justify it.

It would be important to note that sewer bursts can possibly lead to a concentration of the pathogen at the point of origin of the burst.

The following sentence should read as, “It is likely that sewer bursts do not occur at random but are spatially correlated due to underlying weaknesses in the network in those areas. Therefore, targeting the five wards where the majority of sewer bursts were reported could be an efficient way to reduce cholera risk relative to repairing sewer bursts across the city. This is supported by our data, that a high density of bursts were positively associated with cholera cases.”
