# Supplementary material for: A stitch in time: The importance of water and sanitation services (WSS) infrastructure maintenance for cholera risk. A geospatial analysis in Harare, Zimbabwe
Source: PLoS Negl Trop Dis. 2023 Jun 16;17(6):e0011353. doi: 10.1371/journal.pntd.0011353 (PMC10275451; doi:10.1371/journal.pntd.0011353)
Supplement: Attachment — Submitted filename: Response to Reviewers Letter Form_160323.docx [file pntd.0011353.s009.docx]

Dear Reviewers and Editorial Team,

Thank you for taking the time to read and review our revised manuscript “*The importance of Maintenance: Geospatial Analysis of Cholera Risk and Water and Sanitation Infrastructure in Harare, Zimbabwe*”.  We greatly appreciate the thorough work that went into reviewing it. We have worked to address all of the second round of comments received. Please find below the editor and reviewer comments with our responses.

Thank you for your consideration and we hope we have addressed your comments to your satisfaction.

Contents

[1.	Reviewer's Responses to Questions	1](#_Toc128767746)

[2.	Editorial comments and responses	6](#_Toc128767747)

## 1. Reviewer's Responses to Questions

<b>Summary and General Comments</b></br></br>

Use this section to provide overall comments, discuss strengths/weaknesses of the study, novelty, significance, general execution and scholarship. You may also include additional comments for the author, including concerns about dual publication, research ethics, or publication ethics. If requesting major revision, please articulate the new experiments that are needed.

Reviewer #1: (No Response)

Reviewer #3: Thank you for a chance to re-review this manuscript. I appreciate the authors responses to the reviewer and editor comments and believe the paper is much clearer now. However, I have several pending and new comments that need attention:

- The authors do not explain why the data cannot be shared to be consistent with the PLOS Data Policy. Pointing to someone in the Ministry of Land for non-identifiable data is not a sustainable solution to this as a single person rarely will stay in this type of position and there is no clear criteria by which requests for data will be assessed. This includes data on pipe bursts, admin zone boundaries etc. I do not expect GPS points of case households to be shared but many other data sources should be shared. Furthermore, points can be jittered or aggregated (as I doubt the address data were exact). This is really important to allow for reproducibility of results.

In the supplementary material, we have now provided a table outlining the different datasets that were used in the analysis, and their access requirements in the Methods section under the description of each dataset used. Below is a more detailed explanation for reviewers.

After consultations with and by our Zimbabwean counterpart and co-author on the project, we can confirm the data sources that can be shared as part of this publication and others that are proprietary, and third party can be made available on request. In the GitHub repository (for which the link has now been corrected) <https://github.com/dime-worldbank/geospatial-cholera-modelling-zim/tree/main/data> we have made the following datasets available: the derivative estimates of the proportion of households with piped water and proportion with unimproved water as well as proportion with different sanitation risk scores calculated from DHS, modelled using a multinomial GAM. The code is also provided for this estimation process. The input data is publicly available from the Demographic and Health Survey website, if a request is made for it. Please see <https://dhsprogram.com/data/using-datasets-for-analysis.cfm> for further instructions. The code for the sanitation risk index was developed in Stata and is also provided in the code section of the repository.

Auxiliary global datasets used in the analysis, specifically night-time light data, population density data and proximity to rivers are made available in the GitHub repository.

The following datasets are proprietary to a third party and requests for access will be evaluated on a case-by-case basis. Requests should include a summary of the project and description of the nature of the request. Shapefiles for the water and sewer network of Harare, water quality testing data and digitized sewer network complaints are proprietary to City of Harare Water Department and are not usually made open source for security reasons. However, they can be available upon request to [cohweb@hararecity.co.zw](mailto:cohweb@hararecity.co.zw) . The location of private boreholes is managed by Zimbabwean authority ZIMWA. This information will be indicative of households own water sources and would have to be made available on request after evaluation on a case by case basis. Requests can be made via [callcentre@zinwa.co.zw](mailto:callcentre@zinwa.co.zw) . Finally Call Data Records were provided to the World Bank by Econet under a formal Data Sharing Agreement. Requests for the derivative product of the World Bank analysis which was used in this analysis can be made to the World Bank Microdata repository (<https://microdata.worldbank.org/index.php/catalog/?page=1&ps=15>) where it is being uploaded. In the event that other researchers have any delay in receiving a response from these third parties, the authors are happy to follow up on their behalf.

We hope this provides sufficient information to be approved under PLOS NTD Data policy.

| Data Source | Public/Proprietary | Make available on Github? (Y/N) |
| --- | --- | --- |
| DHS 2015 & 2018 [subset pertaining to our analysis] – household access to toilets and drinking water sources and generated index and code | Public and available on GitHub <https://github.com/dime-worldbank/geospatial-cholera-modelling-zim/tree/main/data> | Y |
| Population density data from Facebook Humanitarian Data exchange | Public and available on GitHub | Y |
| Night time lights | Public and available on GitHub | Y |
| Population density (WorldPop) | Public and available on GitHub | Y |
| Proximity to River | Public and available on GitHub | Y |
| Groundwater vulnerability risk index | Proprietary to World Bank, but has been shared on Github | Y |
| Distance to medium sized markets | Public and available on GitHub | Y |
| Water and sewer network shapefile | Proprietary to Third Party (City of Harare). Can be made available on request to [cohweb@hararecity.co.zw](mailto:cohweb@hararecity.co.zw) | N |
| Water quality data | Proprietary to Third Party (City of Harare). Can be made available on request to [cohweb@hararecity.co.zw](mailto:cohweb@hararecity.co.zw) | N |
| Digitized sewer network bursts complaints | Proprietary to Third Party (City of Harare). Can be made available on request to [cohweb@hararecity.co.zw](mailto:cohweb@hararecity.co.zw) | N |
| CDR – Mobility data | World Bank Microdata repository (<https://microdata.worldbank.org/index.php/catalog/?page=1&ps=15> ) | N |
| 1848 private boreholes | Proprietary to third party ZIMWA and can be made available on request to [callcentre@zinwa.co.zw](mailto:callcentre@zinwa.co.zw) | N |
| Georeferenced cholera case data | Original is proprietary to the Ministry of Health and Child Care, Zimbabwe. Can be requested to [pr@mohcc.gov.zw](mailto:pr@mohcc.gov.zw) . Dummy replacements have been provided in the Github | Y |

- In my previous review I had asked about the rational for using different covariates and the authors explained it was based on previous hypotheses and literature (which is now clarified in the text). One missing bit is the geologic vulnerability. What was the thought behind this? In the discussion the authors suggest that they don’t have a plausible mechanism for this relationship so I am not sure I understand why they decided to include it in the first place.

The geological vulnerability layer was generated by the company ZIVA on a subcontracted activity from the World Bank. A report that they provided on the generation of that layer can be provided on request separately. The vulnerability index constructed there included factors such as depth to groundwater, topographical wetness, fissure flow, geological faults and soil permeability. Each of these factors, both individually and in conjunction with one another will affect the ability and speed of contaminated water flow below ground. Given that many households do rely on groundwater for drawing water through wells when the network supply is unreliable, this would definitely have repercussions on cholera risk if they overlapped spatially with areas that experienced high levels of contamination. If the boreholes are drilled deeper than the flow of contaminated water, the risk of contamination may be reduced if the well is considered ‘protected’ due to the lining of the well. However, all the factors mentioned will come into play to determine both how deep contamination may infiltrate the groundwater, and how likely it will be to spread across distances. More information on groundwater flows can be found in (Focazio et al. 2002)

- In response to my previous question about case definitions the authors responded with: “For the WHO case definition provided, are areas where cholera is not known to be occurring neighborhoods? Or is Harare treated as a unit and the suspected case definition is really just ≥ 2 y/o with AWD (except for the first case)?” The authors simply cut and paste the GTFCC recommended case definitions. This does not answer the question of how this was done in Harare. In the methods section the authors should be explicit about what the Ministry of Health used as case definitions in this outbreak exactly not just what WHO/GTFCC recommends. Would also be useful to mention how many cholera cases were culture confirmed during this outbreak.

This information on the Zimbabwe Ministry of Health’s own case definitions has been added to the case data section in lines 178 – 183.

- The authors created a sanitation risk score from 0-1 based on the JMP sanitation ladder but it is not clear from the Methods how exactly this was done. Is each rung of the ladder equally spaced? Why was this done rather than a multinomial like what was done for water indicators? Please update methods to clarify.

The dofile for the creation of the sanitation risk index has now been uploaded to the Github link. The generation of the risk index can be seen in lines 137-160 of the dofile. It is first generated as a categorical index which mirrors the Joint Monitoring Program representation of the sanitation ladder. The rankings are equally spaced with the lowest risk of 0 being those households who reported having an unshared sewered connection OR an unshared VIP latrine, pit latrine with slab or septic tank. A risk level of 1 was given to households who had either of the former two technology types but with a shared sanitation facility due to risk of contamination; those who reported unimproved sanitation facilities had a risk score of 4, and those who reported open defecation had the highest risk score of 5. These categorical values were then normalized to create 166 unique values, and rescaled to lie between 0.01 and 0.99 for modeling purposes. Modeling was conducted using a Beta regression.

- The coefficients in Table 1 remain uninterpretable to the general reader. I realise it is difficult to interpret these but this is indeed a public health journal and general readers will need to be able to map these numbers to some interpretation (especially if you chose to focus on them as a table). I encourage the authors to spend some time thinking about how these could be transformed into interpretable results that could possibly be used in decision making and policy.

An additional table (Table 2) has been added to show model outputs in a more interpretable manner. If we just exponentiate the coefficient, we get an (incidence) rate ratio. However, the rate ratio shows the relative increase in incidence for every unit increase in the covariate. If a covariate ranges from 0 – 0.1 it is difficult to interpret how the risk factor acts on the population (the same is true if the covariate ranges from say 0-1000). So for each covariate we have calculated the range of values experienced by populated grid cells and then split that range into 10 equal strata. We then then calculated the RR between strata – e.g. if the prevalence of piped water goes from 0.2 – 0.6 (range of 0.4), then calculated the RR for every increase of 0.04. Hopefully this this allows the reader to understand the relative risk experienced by different populations better.

Minor:

- Pg 6 - “Finally, a groundwater vulnerability index was developed by Ziva, based on a combination of geologica..” What is Ziva? This has been added to the text

- The track changes version of the manuscript uploaded does not highlight all changes made and it is really hard to assess differences. Thank you for highlighting, this has been revised

- Figure 4: I suggest transforming the axes given that most of the data are less than 100. With current scale its very hard to see the majority of error. Thank you for highlighting, this has been revised

- No reference to GitHub page within manuscript. Please add this to the text. Thank you for highlighting, this has been revised

- Provide inferred flux matrices in GitHub repo. We are unclear what this refers to. If this refers to the human movement data, these data can be shared only on request as explained in the data table. See reviewer 1, question 1.

- The authors responded to my query about the time frame of sewer burst data but did not add this to the paper. Please add that the sewer burst data used in the model was from Dec 2017 through Dec 2018. Please see line 241

- Clarify in limitations that these were based on suspected, not confirmed cholera cases and how this could impact results. Please see line 561

- Can the authors comment on whether illegal connections to the piped water system may compromise its integrity (sometime creating negative pressure)? I know this is an issue in some places but not sure about Harare.

The team’s author who works closely with the City of Harare Water department confirms that yes there are also illegal connections that can cause negative pressures. However, those working closely with the utility report that intermittent supply is the main cause of pressure surges, compromising system integrity. Vandalism outstanding bursts and aging infrastructure also serve to exacerbate maintenance challenges. This information has been added to the manuscript in line 346

- Pg 9, “Covariates were retained in the model if their inclusion led to a decrease in AIC of >2.” Is this through some forward/backward selection process? Please specify in text. Backward, the detail has been added to the text L382

- Double check text that there are no references to the now deleted scenario analyses.

The scenario analysis referenced on line 149, line 520, line 555 and Figure 8 refers to the scenarios which are the spatially targeted scenarios on reducing cholera risk. We have removed the other scenario analysis references.

## 2. Editorial comments and responses

**Global comments:**

Please use line numbers in the future. Thank you for highlighting, this has been revised

Please double check spacing throughout the manuscript. Some areas are missing spaces, some are double-spaced where they should not be. Thank you for highlighting, this has been revised

Please break down run on sentences. Thank you for highlighting, this has been revised where indicated

Please spell out numbers from zero to nine. Thank you for highlighting, this has been revised

Do not use symbols, i.e, < or >. Please spell out the words less than or greater than. Thank you for highlighting, this has been revised

**Minor Comments**

**Abstract:** One run-on sentence should be broken down, instead reading as: One possible explanation for this observation is that sewer bursts led to the contamination of the piped water network. This turned access to piped water, usually assumed to be associated with reduced cholera risk, into a risk factor itself. Such events highlight the importance of maintenance in the provision of SDG improved water and sanitation infrastructure. Thank you for highlighting, this has been revised

**Introduction:** Vibrio cholerae is the etiological agent of cholera, not the etiological agents. Also, serotypes O1 and O139 are responsible for causing cholera, as there are well over 200 serotypes characterized to date. Please specify this in the introduction, and cite. Thank you for highlighting, this has been revised

Salmonella Typhi should be written as, “*Salmonella enterica* serovar Typhimurium” Thank you for highlighting, this has been revised

The sentence, “In particular, outbreaks in developing country contexts are usually attributed to where households have low access to ‘improved’ water and sanitation infrastructure such as networked water and sewer systems (as defined by the WHO Joint Monitoring program) [3], [21]” is somewhat unclear. What is meant by contexts? Please clarify. Thank you for highlighting, this has been revised

For the phrase developing country, it is usually more appropriate to utilize the term, “emergent nation”. Thank you for highlighting, this has been revised

The phrase well documented should have a hyphen between the words. Thank you for highlighting, this has been revised

**Study Area:** It is uncertain what the acronym DHS stands for. Please spell out prior to using the acronym in the paper. Thank you for highlighting, this has been revised

**Case Data:** “Age and gender of the patients is” should be changed to “Age and gender of the patients are” Thank you for highlighting, this has been revised

How many cases were duplicates that were removed? Please specify. Thank you for highlighting, this has been revised

**Population Characteristics Data:** What does the HDX stand for after Facebook? Please specify the acronym as requested previously. Thank you for highlighting, this has been revised

“Household access to water, sanitation and hand washing infrastructure was taken from the DHS 2015.” Was should be changed to were. Thank you for highlighting, this has been revised

The single sentence: “It placed households reporting their facility to be a flush toilet connected to a piped sewer system and not shared at the top of the ladder and therefore lowest risk, then flush toilets connected to a septic tank, pit latrine, VIP latrine or pit latrine with slab that were unshared; then each of the former facility types that were shared; then unimproved sanitation facilities; and finally households reporting open defecation as those with the highest sanitation risk.” Should be broken down into multiple sentences. Thank you for highlighting, this has been revised

**Spatial modeling:**

The word “the” should be placed before Poisson. Have changed to ‘a’

The following sentence should read as, “For onsite facilities, contamination can occur through unlined or poorly constructed pit latrines which may seap content into the groundwater supply. Thus, groundwater vulnerability is also included in the model because it feeds into the many borewells in the city[41].” Thank you for highlighting, this has been revised

The following paragraph should read as, “We include the market and public facility locations, as well as the population density data, due to the pathways to contamination that have been identified in contaminated food and poor personal hygiene [11]. In addition, cholera transmission has been associated with case clusters among close contacts respectively [14]. We finally include an indicator that accounts for mobility by calculating the expected imported cases that come in from other areas. Emigration could lead to the generation of additional cholera cases in an area, given research showing that population mobility can lead to spread of cholera to new areas [16], [18]–[20]. Thank you for highlighting, this has been revised

On page 8, please clarify the acronym SIM. Thank you for highlighting, this has been revised

The following sentence should read as, “For every individual/SIM we calculated the probability that an individual traveling to a destination became infected in the last ten days. This calculation was based on the 1) locations the individual spent time in during those ten days, 2) duration of time spent in each location and 3) local level of risk at each location (estimated by calculating observed weekly incidence per tower catchment). Thank you for highlighting, this has been revised

**Results**

All instances of the phrase, ‘the modeling’ should be changed to ‘the models.’ Thank you for highlighting, this has been revised

The sentence, “In addition, there is undoubtedly error in the predicted proportion with unimproved water as these data are based on a small number of obfuscated DHS data points which could lead to a spurious relationship.” Is unclear. This seems like an important point, please expand, but be careful not to jump to conclusions without data to justify it. Thank you for highlighting, this has been revised

It would be important to note that sewer bursts can possibly lead to a concentration of the pathogen at the point of origin of the burst. Thank you for highlighting, this has been revised

The following sentence should read as, “It is likely that sewer bursts do not occur at random but are spatially correlated due to underlying weaknesses in the network in those areas. Therefore, targeting the five wards where the majority of sewer bursts were reported could be an efficient way to reduce cholera risk relative to repairing sewer bursts across the city. This is supported by our data, that a high density of bursts were positively associated with cholera cases.” Thank you for highlighting, this has been revised
